# Single-Port vs. Multi-Port Robotic Surgery in Urologic Oncology: A Comparative Analysis of Current Evidence and Future Directions

**DOI:** 10.3390/cancers17172847

**Published:** 2025-08-29

**Authors:** Stamatios Katsimperis, Lazaros Tzelves, Georgios Feretzakis, Themistoklis Bellos, Konstantinos Douroumis, Nikolaos Kostakopoulos, Andreas Skolarikos

**Affiliations:** 1 Second Department of Urology, National and Kapodistrian University of Athens, Sismanogleio Hospital, 15126 Athens, Greece; 2 School of Science and Technology, Hellenic Open University, 26335 Patras, Greece; 3 Department of Urology, Red Cross General Hospital of Athens, 11526 Athens, Greece; 4 First Department of Urology, National and Kapodistrian University of Athens, 11527 Athens, Greece; 5 First Department of Urology, Metropolitan General Hospital, 15562 Athens, Greece

**Keywords:** single-port robotic surgery, multi-port robotic surgery, da Vinci SP, urologic oncology, robot-assisted radical prostatectomy, robot-assisted partial nephrectomy, robot-assisted radical cystectomy, minimally invasive surgery, surgical outcomes, learning curve, oncologic surgery innovation

## Abstract

The evolution of robotic surgery in urologic oncology has led to the emergence of single-port (SP) robotic systems as a potential alternative to the widely adopted multi-port (MP) platforms. This narrative review provides a comprehensive comparison between SP and MP robotic systems, the former of which received FDA approval in 2018 and CE marking in 2024, focusing on their application across radical prostatectomy, partial and radical nephrectomy, and radical cystectomy. Drawing from the most current literature, we examine perioperative outcomes, oncologic efficacy, postoperative recovery, and complication rates. The review highlights the technical challenges unique to SP surgery, including restricted triangulation, limited instrumentation, and a defined learning curve, while also emphasizing innovations such as transvesical prostatectomy and the Supine Anterior Retroperitoneal Access (SARA) approach. Additionally, we explore the potential impact of emerging technologies—such as artificial intelligence, augmented reality, and telesurgery—on the future of SP platforms. Despite early limitations, SP systems have demonstrated comparable safety and effectiveness in selected cases and may offer unique advantages in specific anatomical scenarios. Continued innovation, structured training, and robust long-term outcome data will be essential for the broader adoption and integration of SP robotic surgery in clinical practice.

## Simple Summary

Robotic-assisted surgery has transformed the management of urologic cancers, traditionally relying on multi-port systems that require multiple incisions. More recently, single-port platforms have emerged, offering the potential to minimize surgical trauma by using a single incision. This narrative review compares single-port and multi-port robotic approaches in radical prostatectomy, nephrectomy, and cystectomy. We summarize current evidence on perioperative outcomes, oncologic safety, functional results, and complication rates. While single-port systems show comparable oncologic outcomes to multi-port platforms in selected patients, they may offer advantages such as reduced postoperative pain, shorter hospital stays, and improved cosmesis. However, limitations include reduced instrument triangulation, restricted instrumentation, and a defined learning curve. Innovations like the transvesical approach and novel retroperitoneal access techniques further highlight the adaptability of single-port systems. Looking ahead, integration of technologies such as artificial intelligence, augmented reality, and telesurgery may enhance the precision and accessibility of single-port robotic surgery. Ongoing technological refinement, structured training, and long-term outcome data will be essential for wider adoption in clinical practice.

## 1. Introduction

The emergence of robotic surgery has revolutionized the management of urologic cancers by introducing enhanced precision, reduced invasiveness, and improved perioperative outcomes. Among the earliest milestones was the adoption of the da Vinci multi-port (MP) platform, which rapidly became the gold standard in robotic-assisted urologic procedures including radical prostatectomy, partial nephrectomy, and radical cystectomy. Currently, over 85% of radical prostatectomies in the United States are performed using robot-assisted techniques [1,2]. Similarly, the adoption of robotic platforms is increasingly prevalent in other complex urologic procedures, such as upper tract surgeries [3]. These procedures benefited from the platform’s enhanced dexterity and three-dimensional visualization, which together improved oncological and functional outcomes while reducing surgical morbidity [4,5]. Despite these advancements, MP systems come with limitations such as the need for multiple incisions, the risk of external arm collisions, increased setup complexity and difficulty when working in small spaces.

In response to these concerns and the drive toward even less invasive methods, the da Vinci single-port (SP) robotic system was developed and received Food and Drug Administration (FDA) approval in 2018 and from January 2024 became available in Europe, having received CE marking. This platform allows surgeons to perform complex procedures through a single 25 mm port, using a flexible camera and three multi-jointed robotic arms [6]. This design minimizes surgical trauma and optimizes instrument control in confined spaces, making it particularly appealing in urologic oncology where anatomical regions can be deep and narrow. By reducing the number of incisions, single-port surgery improves cosmetic outcomes and may lower the risk of port-site complications. The transition from MP to SP technology has sparked considerable interest and investigation, particularly regarding whether these new platforms maintain equivalent oncologic safety, offer improved perioperative outcomes, and provide tangible benefits in patient recovery and satisfaction. Multiple studies, including randomized trials, prospective cohorts, and meta-analyses, have been conducted to compare these platforms. In this structured narrative review with systematic synthesis of comparative outcomes, we synthesize findings from several recent and high-quality sources to provide a comprehensive comparative analysis of SP and MP robotic platforms in urologic oncology. Our review will cover perioperative and functional outcomes, oncologic efficacy, disease-specific applications, as well as challenges and future directions. The aim is to present a balanced, evidence-based perspective on the current status and future role of SP robotic systems relative to MP platforms in urologic cancer surgery.

## 2. Clinical Applications in Prostate, Kidney, and Bladder Cancer

The clinical applications of robotic-assisted surgery in urologic oncology span three primary malignancies: prostate, kidney, and bladder cancer. Each of these areas has seen significant innovation with the adoption of SP platforms, with varying degrees of penetration and data availability.

### 2.1. Radical Prostatectomy

Prostate cancer represents the most extensively studied domain for SP robotic surgery. Single-port robot-assisted radical prostatectomy (SP-RARP) has emerged as a viable and increasingly utilized technique in the surgical management of localized prostate cancer. Since Kaouk et al. first reported successful SP-RARP procedures with minimal complications and discharge within 24 h, the platform has garnered attention for its potential to minimize invasiveness without compromising oncologic safety [7,8]. Various approaches—including transperitoneal, extraperitoneal, Retzius-sparing, and transvesical—have been adapted for SP-RARP, each offering unique perioperative advantages (Table 1) [2,9,10,11]. The SP system’s single-site access lends itself particularly well to extraperitoneal, Retzius-sparing and transvesical techniques, where preservation of the peritoneum may support quicker recovery and fewer gastrointestinal complications. Comparative studies from multiple institutions have consistently shown that SP-RARP achieves oncologic outcomes comparable to those of multi-port RARP (MP-RARP). While early experiences with SP-RARP suggested a potentially higher rate of positive surgical margins (PSM), likely reflecting the learning curve associated with the new platform, more mature series have demonstrated comparable PSM rates between SP and MP approaches [12,13,14]. In one relatively large retrospective comparison, Vigneswaran et al. found no statistically significant difference in PSM rates between the two platforms, reinforcing the oncologic equivalence of SP-RARP when performed by experienced surgeons [15]. Similar findings were reported by Moschovas et al., who also observed comparable PSM outcomes between SP and MP procedures, even after adjusting for tumor stage and surgical technique [9]. These results have been further supported by both retrospective and prospective cohorts, including propensity score-matched analyses by Noh et al. (PSM 19.4% in both groups) and Ju et al. (0% in both groups) [10,16]. Notably, oncologic equivalence between SP and MP platforms appears to be preserved even in high-risk patients and cases with extracapsular extension. In a multi-institutional study by Huang et al., the difference in PSM rates between SP and MP approaches was not statistically significant (31.3% vs. 24.5%, *p* = 0.08). Similarly, Moschovas et al. reported a higher incidence of extraprostatic extension in the SP cohort without a corresponding increase in PSM rates, with a difference of just 1.4% between the SP and Xi groups (95% CI: −11% to 14%) [9,17]. These findings are further corroborated by a recent meta-analysis by Ge et al., which included six studies encompassing 1451 patients and found no significant difference in PSM rates between SP and MP platforms (OR = 0.95, 95% CI [0.74–1.22], *p* > 0.05), with low heterogeneity across studies (I^2^ = 48.5%) [18].

The role of lymph node dissection remains particularly relevant in prostate cancer surgery, especially when using SP robotic platforms. While lymphadenectomy has been successfully performed with the SP system, several studies—including those by Vigneswaran, Huang, Shiang, and in the meta-analysis by Nguyen et al.—have reported a trend toward slightly lower lymph node yields in SP cases [15,17,19,20]. This observation appears to stem more from differences in surgical planning, surgeon experience, and patient risk stratification than from limitations of the platform itself. Notably, Harrison et al. observed reduced nodal yields specifically in extraperitoneal SP-RARP compared to standard MP-RARP, further highlighting the influence of approach selection on outcomes [21]. This discrepancy was attributed by the authors, at least in part, to a higher likelihood of extended lymph node dissection in the MP cohort, as these patients often presented with more advanced disease. It seems that surgeons may choose a more limited dissection during the early learning curve or in patients with low-risk disease profiles. Nevertheless, certain technical considerations, such as the lack of a dedicated assistant port or the use of a transvesical approach, may require procedural adaptations and could modestly influence the extent of dissection in select cases, as noted by Soputro et al. [22]. Taken together, the variability in reported lymph node yields between SP and MP platforms appears to arise less from inherent limitations of the SP system and more from contextual factors. Differences in surgical planning, surgeon experience, and patient risk stratification strongly influence the extent of dissection. Reduced nodal yields reported in extraperitoneal SP-RARP, for instance, likely reflect the influence of approach selection and disease stage, as MP cohorts were more likely to undergo extended lymphadenectomy in higher-risk cases. Surgeons early in their SP experience may also favor more limited dissections, particularly in low-risk patients. In addition, certain technical considerations—such as the absence of a dedicated assistant port or the use of transvesical access—may modestly constrain dissection in select scenarios.

Perioperative outcomes are where SP-RARP has shown the most consistent advantages. Multiple studies, including meta-analyses by Li et al. and reviews by Wei et al., have found that SP-RARP is associated with shorter hospital stays, reduced catheterization times, and decreased postoperative opioid use [23,24]. Specifically, Li et al. reported a significantly shorter hospital stay with SP-RARP (weighted mean difference [WMD] −17.86 h, 95% CI −27.80 to −7.92; *p* = 0.0004), along with a reduction in catheterization time (WMD −1.51 days, 95% CI −2.60 to −0.41; *p* = 0.007). The odds of postoperative opioid use were also significantly lower (OR 0.26, 95% CI 0.13 to 0.53; *p* = 0.0002), and notably, patients undergoing SP-RARP were far more likely to require no pain medication during hospitalization (OR 14.41, 95% CI 5.22 to 39.76; *p* < 0.00001) [24]. These outcomes are especially notable in extraperitoneal and transvesical approaches, which reduce bowel manipulation, pneumoperitoneum, and Trendelenburg requirements, as highlighted by Zeinab et al. [2] Abaza et al. reported same-day discharge in 88% of their SP-RARP patients, attributing this success to reduced postoperative pain and accelerated recovery facilitated by a single-incision approach [25]. Vigneswaran et al. further supported these findings by documenting lower pain scores on postoperative day one in SP patients [15]. Reduced blood loss has also been reported with SP-RARP compared to MP-RARP; however, the difference—typically around 50 mL—is modest and its clinical relevance remains uncertain [9,17,19,22,26]. Regarding operative times, although some early comparisons, such as those by Harrison et al., suggested longer operative times for SP-RARP due to the learning curve, the difference was typically a little above 30 min and tended to decrease with growing surgical experience [21].

Functional outcomes, particularly urinary continence and erectile function have also been evaluated. Most studies indicate parity between SP and MP platforms at three to six months postoperatively, including those by Moschovas et al., Ju et al., and Lenfant et al. [9,16,26]. Noh et al. reported a lower rate of complete nerve sparing in SP-RARP, which they attributed to reduced tissue traction capabilities of the SP instruments [10]. Nonetheless, Harrison et al. observed better erectile function recovery at six months in the SP-RARP group, likely due to refined dissection and reduced manipulation achieved with the SP-platform [21]. Across studies, continence rates were comparable at early and intermediate follow-up intervals, with Kaouk et al. and Zhou et al. reporting early return of continence within days following transvesical SP-RARP [11,27].

**Table 1:** Key studies on single-port robotic radical prostatectomy.

Author	Year	Journal	Description	Study Design	Sample Size
Kaouk et al. [7]	2014	European Urology	First clinical SP-RARP study	Prospective case series	19
Abaza et al. [25]	2020	Journal of Endourology	Comparison of adoption methods by two surgeons	Prospective cohort	74
Moschovas et al. [9]	2021	European Urology	SP vs. MP-RARP comparison	prospective cohort with propensity score matching	142
Noh et al. [10]	2022	Journal of Endourology	SP vs. MP-RARP comparison	Retrospective cohort	169
Abou Zeinab et al. [2]	2023	Urology	Extraperitoneal vs. transperitoneal SP-RARP	Retrospective cohort	476

### 2.2. Partial and Radical Nephrectomy, and Nephroureterectomy

In the management of upper urinary tract malignancies, SP robotic platforms have been successfully applied to partial nephrectomy (PN), radical nephrectomy (RN), and, more recently, to nephroureterectomy (Table 2). However, given the function-preserving nature of PN and the potential ergonomic (reduced instrument clashing), cosmetic, and recovery-related advantages of the SP approach, the majority of published clinical studies have focused on SP robotic partial nephrectomy (SP RAPN).

Kaouk and colleagues reported one of the earliest series of SP-RAPN, demonstrating feasibility and acceptable warm ischemia times (WIT) (average WIT = 25 min) using the retroperitoneal approach [28]. Since then, many studies comparing SP RAPN with multi-port robotic partial nephrectomy (MP RAPN) have been published demonstrating comparable perioperative outcomes, including operative time, estimated blood loss (EBL), ischemia time, length of hospital stay (LOS) and complication rates. However, the tumors treated with SP techniques are typically less complex, reflecting cautious adoption during the early phase of the learning curve. The first of these studies was a large multicenter, propensity score-matched analysis conducted by Okhawere et al. on behalf of the Single Port Advanced Robotics Consortium (SPARC), in which 146 SP RAPN were compared with 146 MP RAPN [29]. Matching was performed based on variables such as age, gender, laterality, body mass index (BMI), nephrometry score, hypertension, and diabetes status. Prior to matching, the SP group presented with lower tumor complexity—reflected in a smaller average tumor size (2.93 vs. 3.42 cm) and lower median R.E.N.A.L. scores (6 vs. 7). Post-matching, both cohorts were balanced in tumor characteristics, including size and nephrometry score. The study found no significant differences between the two groups in terms of EBL, operative time, complication rates, length of hospital stay LOS, or PSM. Interestingly, although the SP group had a longer mean ischemia time (18.3 vs. 13.8 min), operative time for high-complexity tumors was shorter in the SP cohort (108 vs. 167 min) [29]. These findings suggest that SP robotic PN is a feasible and safe option, particularly in experienced hands, though ischemia time may be prolonged during the early learning curve. In another propensity score-matched study, Harrison et al. compared SP and MP RAPN across 48 SP and 238 MP cases, matching patients based on age, sex, BMI, nephrometry score, and history of prior abdominal surgery [30]. After matching, the study found no significant differences in PSM rates or short-term recurrence at a median follow-up of approximately 19 to 21 weeks. One notable advantage of the SP approach was a significant reduction in postoperative opioid use: median morphine milligram equivalents (MME) on postoperative day 1 were 4.6 in the SP group versus 9.8 in the MP group, and cumulative in-hospital use was also lower (5.1 vs. 9.3 MME). While the SP group had a statistically shorter median LOS (1.4 vs. 1.6 days), this difference seems to be relevant from a statistical point of view rather than from a clinical one. In terms of PSM and recurrence two more studies by Palacios et al. and Mehrazin et al. demonstrated equivalent oncologic outcomes between SP and MP RAPN [31,32]. These findings were further supported by two recent meta-analyses, reinforcing the oncologic equivalence of the two approaches [33,34].

While oncologic outcomes appear comparable between SP and MP RAPN, increasing attention has also been directed toward functional and perioperative parameters—such as EBL, WIT and LOS—which are critical indicators of surgical quality and postoperative recovery. Palacios et al. and Glaser et al. reported no significant difference in EBL between the two approaches, consistent with findings by Okhawere et al., whereas Mehrazin et al. observed a lower EBL in the SP-RAPN cohort [29,31,32,35]. In terms of WIT, Okhawere et al. reported significantly longer WIT in the SP-RAPN group compared to MP-RAPN (mean 18.3 vs. 13.8 min), potentially reflecting the early learning curve and technical challenges associated with the single-port platform [29]. Similarly, two recent meta-analyses supported this finding: Hu et al. reported a weighted mean difference (WMD) of 3.13 min (95% CI: 0.81–5.46; *p* = 0.008), and Nguyen [36] et al. found a mean difference of 4.6 min (95% CI: 2.8–6.3; *p* < 0.001) in favor of the MP approach [33]. In contrast, Palacios et al. found no significant difference in WIT between SP and MP techniques, a result echoed by Lv et al., whose meta-analysis reported a non-significant WMD of 3.01 min (95% CI: −1.32 to 7.34; *p* = 0.17) [31,34].

LOS serves as a commonly used metric to assess recovery after minimally invasive surgery, and its potential reduction has been cited as one of the expected benefits of single-port techniques. Several studies have examined this outcome in the context of SP-RAPN, with varying results. Mehrazin et al. and Okhawere et al. both reported no significant difference in LOS between SP and MP cohorts, while Glaser et al. similarly found that most patients in both groups were discharged on postoperative day one [29,32,35]. In contrast, Palacios et al. observed a shorter LOS among patients undergoing retroperitoneal SP-RAPN compared to retroperitoneal MP-RAPN; however, the SP group had less complex tumors, which may have influenced the result [31]. In another matched analysis, Harrison et al. reported a statistically shorter median LOS for SP-RAPN (1.4 days) versus MP-RAPN (1.6 days), yet the absolute difference of 0.2 days is likely of limited clinical relevance [30].

Further insight comes from two recent meta-analyses with conflicting results. Hu et al. found that SP-RAPN was associated with a statistically shorter hospital stay (WMD −0.26 days; 95% CI: −0.36 to −0.15; *p* < 0.00001), suggesting a modest but consistent benefit [33]. In contrast, Lv et al. reported no significant difference in LOS between SP and MP approaches (WMD −0.23 days; 95% CI: −0.69 to 0.23; *p* = 0.32), highlighting ongoing uncertainty in the pooled evidence [34].

Institutional data add another layer to this discussion. Abaza et al. assessed their experience with the first 100 SP robotic procedures, including 18 partial nephrectomies. Focusing on this subgroup, they reported a significantly higher rate of same-day discharge for SP-RAPN compared to MP-RAPN (83% vs. 17%; *p* < 0.001), despite applying identical outpatient protocols [37]. While this suggests that the SP platform may support earlier discharge in selected patients, it also underscores the importance of institutional practices, perioperative pathways, and surgical experience. Conflicting results on hospital stay likely reflect variations in discharge criteria, enhanced recovery protocols, and case selection rather than a consistent effect of the platform itself. These observations underscore the importance of considering patient, surgeon, and institutional factors when interpreting comparative outcomes between SP and MP systems.

In addition to platform comparisons, the surgical approach itself—transperitoneal versus retroperitoneal—has also been explored within the SP-RAPN setting, as it may influence operative efficiency and access depending on tumor location. The choice between transperitoneal and retroperitoneal access in SP-RAPN is influenced by tumor location, surgeon familiarity, and institutional protocols. Rich et al. compared 84 patients undergoing SP-RAPN, with 44 treated transperitoneally and 40 via the retroperitoneal route [38]. Their analysis showed no statistically significant differences in estimated blood loss (95 vs. 76 mL), warm ischemia time (16.1 vs. 15.6 min), or length of hospital stay (1.33 vs. 1.06 days). Notably, the retroperitoneal approach was associated with a shorter mean operative time (156.4 vs. 190.2 min, *p* = 0.011), potentially due to more direct access to posterior tumors and avoidance of bowel mobilization. Although intraoperative complications and conversion rates were lower in the retroperitoneal group, these differences were not statistically significant.

While SP partial nephrectomy has been the primary focus of most clinical studies due to its function-preserving goals and technical complexity, the application of SP platforms to radical nephrectomy has also been explored. However, the available evidence for SP radical nephrectomy remains more limited, with fewer studies reporting outcomes in this setting. As radical nephrectomy is generally less technically demanding and often performed for larger or more centrally located tumors, the perceived benefits of the SP approach—such as reduced invasiveness or improved cosmesis—must be weighed against its technical constraints. Early institutional experience with SP robot-assisted radical nephrectomy (SP RARN) was reported by Fang et al. in 2019, who evaluated both SP RARN and SP RAPN [39]. Their analysis revealed no significant differences between the two groups regarding tumor size, operative time, EBL, or immediate postoperative complications. Building on this, Berry et al. conducted a matched analysis comparing SP and MP RARN for both high- and low-complexity renal masses, stratified by R.E.N.A.L. nephrometry scores [40]. Among high-complexity tumors (score ≥ 7), SP surgery was associated with significantly longer operative times (248.4 vs. 188.1 min, *p* = 0.02) but a shorter LOS (1.9 vs. 2.8 days, *p* = 0.02) compared to MP surgery [40]. For low-complexity cases, operative time, EBL, and LOS were similar between the two approaches. These findings suggest that while SP surgery is feasible for complex cases, it may require additional operative time, likely reflecting technical adjustments during the learning curve. Similarly, Okhawere et al., using data from the SPARC, conducted a propensity score–matched comparison of 91 SP and 91 MP RARN [41]. Their results demonstrated no significant differences in operative time (175 vs. 170 min), EBL (100 vs. 125 mL), LOS (1.3 vs. 1.4 days), or complication rates (11% vs. 13%), with similar oncologic outcomes across both groups [41].

Beyond nephrectomy, SP robotic platforms have also been applied to more complex upper tract procedures such as radical nephroureterectomy (RANU), though clinical experience in this setting remains limited. Nephroureterectomy with en bloc excision of the ipsilateral ureteral orifice and bladder cuff remains the standard treatment for upper tract urothelial carcinoma (UTUC). As surgeon experience with the da Vinci SP platform has grown, single-port robotic nephroureterectomy (SP-RANU) has been increasingly adopted using both transperitoneal and retroperitoneal approaches. However, the literature describing outcomes remains limited, and comparative studies with multi-port techniques are lacking. Traditional multi-port RANU can be technically cumbersome due to the need to operate across multiple abdominal quadrants, which historically necessitated patient repositioning and robotic redocking to access the distal ureter and bladder cuff. Although these issues have been mitigated by the da Vinci Xi system, the MP approach still encounters challenges in retroperitoneal surgery, particularly due to instrument clashing and restricted mobility in the confined workspace. The SP platform, with its single-arm design and coaxial access, may overcome some of these ergonomic limitations. Pellegrino et al. described their early experience using both transperitoneal and retroperitoneal SP-RANU, highlighting its feasibility but noting technical challenges—particularly with lymphadenectomy—and emphasizing the need for further study to assess oncologic efficacy [42]. Bang et al. provided one of the most detailed reports to date, presenting a cohort of 20 patients who underwent retroperitoneal SP-RANU [43]. They reported a median operative time of 150.5 min, EBL of 122.5 mL, a median LOS of 4.5 days, and no intraoperative complications. Only one recurrence was observed during a mean follow-up of 180 days [43]. Despite these encouraging early results, there are no comparative studies to determine whether SP-RANU offers advantages over existing approaches. Moreover, key elements of nephroureterectomy—such as bladder cuff management and lymph node dissection—remain technically demanding with the current SP system, and may limit its broader application until further refinements are developed.

**Table 2:** Key studies on single-port robotic upper tract surgery.

Author	Year	Journal	Description	Study Design	Sample Size
Glaser et al. [35]	2022	Journal of Robotic Surgery	SP vs. MP-RAPN outcomes and analgesia	Retrospective cohort	78
Rich et al. [38]	2023	European Urology Focus	Transperitoneal vs. retroperitoneal SP-RAPN	Prospective cohort	219
Fang et al. [39]	2020	Journal of Robotic Surgery	Initial experience with SP-RAPN and SP-RARN	Retrospective case series	16
Bang et al. [43]	2023	Journal of Clinical Medicine	SP retroperitoneal RANU	Retrospective case series	20
Pellegrino et al. [44]	2023	European Urology	SARA technique for SP renal surgery	Prospective cohort	18

### 2.3. Radical Cystectomy

Single-port robot-assisted radical cystectomy (SP-RARC) has emerged as a technically feasible approach, building on the advantages of the SP platform for multi-quadrant pelvic surgery—namely enhanced maneuverability, fewer incisions, and improved access for both cystectomy and urinary diversion (Table 3). Early feasibility was demonstrated by Kaouk et al. and Zhang et al., who independently reported successful completion of SP-RARC with intracorporeal urinary diversion in small cohorts of four patients, without conversions or intraoperative complications [45,46]. In Zhang’s series, mean operative time was 270 min, with average blood loss of 250 mL, lymph node yield of 12.5, and hospital stay of 5.5 days [46]. Only one patient experienced a Clavien II complication (blood transfusion), with no major events within 90 days postoperatively. Comparative data between SP and MP platforms are still limited but growing. Gross et al. conducted a 1:2 propensity-matched analysis and found equivalent outcomes in terms of EBL, operative duration, complication rates, and positive surgical margins [47]. However, the SP group had a significantly lower lymph node yield (11.9 vs. 17.1, *p* = 0.035), raising concerns about oncologic staging [47]. Ali et al., in a comparison of 14 SP and 20 MP intracorporeal cystectomies, also found no significant differences in most perioperative parameters, but noted significantly less postoperative narcotic use (11.5 vs. 25 MME, *p* = 0.047) and faster return of bowel function (2 vs. 3 days, *p* = 0.032) with the SP approach [48].

In the largest series to date, Fang et al. evaluated 96 patients and found that SP-RARC was associated with shorter operative time (386 vs. 454 min, *p* < 0.01) and quicker bowel recovery (3.4 vs. 4.5 days, *p* < 0.01), while complication rates, readmissions, and positive margin rates remained comparable between groups [49]. Despite promising short-term outcomes, a consistent observation across studies is the lower lymph node yield with the SP platform, raising questions about its oncologic adequacy, particularly in patients requiring extended pelvic lymph node dissection. Reports of lower lymph node yields with SP radical cystectomy likely reflect both technical and contextual factors. The confined working space, reduced triangulation, and absence of a dedicated assistant port may modestly limit the extent of extended pelvic lymphadenectomy. In addition, during the early adoption phase, surgeons may opt for more conservative dissections, particularly in lower-risk patients. These factors suggest that the observed discrepancy is less an inherent limitation of the SP platform than a product of technical adaptation and surgeon experience. Altogether, these findings support SP-RARC as a safe and viable alternative to MP-RARC, especially in select patients with limited abdominal space or prior surgery. However, the lower lymph node yield and lack of long-term oncologic data underscore the need for further multicenter prospective studies to fully validate its role in bladder cancer surgery. While preliminary data suggest comparable perioperative and oncologic outcomes between SP and MP radical cystectomy, including early experiences with intracorporeal urinary diversion, long-term survival outcomes and functional recovery remain insufficiently reported. The limited adoption of SP for cystectomy underscores the need for further multi-institutional and prospective data before definitive conclusions can be drawn.

To provide an integrative overview, the main comparative findings between SP and MP platforms across perioperative outcomes, oncologic efficacy, postoperative recovery, and complication rates are summarized in Table 4.

## 3. Technical Challenges and Learning Curve

The transition from MP to SP robotic systems in urologic oncology introduces a spectrum of unique technical challenges and a clearly defined learning curve that must be addressed for successful clinical implementation. While the SP platform offers theoretical advantages—such as reduced instrument clashing, improved cosmesis, and the potential for faster recovery—its effective use depends critically on surgeon familiarity, tailored procedural strategies, and institutional training infrastructure.

One of the most significant technical limitations in SP surgery stems from the lack of instrument triangulation. Unlike MP platforms, where instruments are inserted at varying angles to allow natural countertraction, the SP system channels all instruments and the camera through a single port. This single-axis configuration alters instrument dynamics and restricts maneuverability, particularly during complex steps such as dissection and renorrhaphy. Although the SP platform incorporates fully wristed tools with en-bloc rotation and the “cobra” mode camera to partially offset these constraints, the unique ergonomics demand visual-spatial reorientation and reconditioning of the surgeon’s muscle memory. These challenges are further compounded by the absence of some key instruments. Tools such as the ProGrasp™ forceps, Vessel Sealer, and EndoWrist Stapler (all by Intuitive Surgical, Sunnyvale, CA, USA) are currently unavailable for SP use, and near-infrared fluorescence imaging (e.g., Firefly^®^) (Intuitive Surgical, Sunnyvale, CA, USA) has not yet been fully integrated into the platform. This limited instrument armamentarium directly impacts case selection and procedural planning, particularly for surgeries involving advanced dissection or reconstruction. The absence of these instruments has tangible clinical implications. For example, the lack of an EndoWrist Stapler limits options for rapid vascular control and bowel anastomosis, requiring surgeons to rely on alternative suturing or clipping methods. Similarly, without a Vessel Sealer, hemostasis must often be achieved through bipolar cautery or conventional ligation. In partial nephrectomy, these constraints necessitate adaptations in renorrhaphy technique, while in cystectomy, they may complicate intracorporeal urinary diversion and bowel reconfiguration. Moreover, the incomplete integration of fluorescence imaging reduces the ability to perform real-time vascular mapping, which may affect intraoperative decision-making.

SP robotic surgery also necessitates precise considerations regarding access and port placement. Typically, a 2–2.5 cm fascial incision is required, often created using the Hasson technique. The system functions optimally when a minimum of 10 cm separates the cannula and the target anatomy to ensure adequate instrument deployment and full range of motion. This requirement may pose challenges in anatomically constrained spaces like the retroperitoneum or deep pelvis, especially in thin patients or when operating on lower-pole renal masses. When such space is lacking, surgeons often resort to “floating docking,” in which the wound protector seal is elevated to artificially extend the working distance. To overcome these limitations, Pellegrino et al. introduced the Supine Anterior Retroperitoneal Access (SARA) technique, which involves a McBurney incision with the patient in the supine position [44]. This innovation simplifies retroperitoneal entry while avoiding the physiological strain of lateral decubitus positioning and reducing pneumoperitoneum-related complications. Early outcomes with SARA demonstrated feasibility, safety, high trifecta rates in partial nephrectomy, and minimal postoperative pain, particularly in patients with significant comorbidities or prior abdominal surgeries.

Another technical barrier lies in the constrained role of the assistant during SP procedures. The lack of a dedicated assistant port restricts suction, retraction, and instrument exchange. Workarounds include the use of flexible suction catheters, such as the ROSI system, or placing an auxiliary assistant port adjacent to the main SP cannula. However, such adaptations often face spatial limitations and risk compromising the efficiency of intraoperative assistance. Some surgeons utilize the fourth channel of the SP cannula to accommodate suction if it is not otherwise in use, though this too requires careful coordination.

These challenges become even more apparent in procedure-specific applications. One particularly complex innovation is the SP transvesical radical prostatectomy (SP-TV-RP), a technique introduced by Kaouk et al. that entirely bypasses the peritoneal cavity by accessing the prostate through a suprapubic incision into the bladder [11]. After bladder insufflation and docking, the procedure proceeds intravesically with a posterior bladder neck incision, dissection of the seminal vesicles and vasa deferentia, development of the rectoprostatic plane, anterior bladder neck division, and urethral transection. The operation concludes with posterior reconstruction and urethrovesical anastomosis. This approach minimizes bowel manipulation and may offer certain anatomical advantages but requires exceptional skill due to its unconventional visual orientation and limited working space. It exemplifies how novel access routes within SP surgery can expand procedural possibilities while imposing new ergonomic and technical demands.

SP partial nephrectomy (SP-PN) also requires specific adaptations. The renorrhaphy phase, in particular, is technically more demanding due to the elbow-like articulation of SP instruments. Port placement strategies must consider the anatomical target and spatial limitations, and in retroperitoneal approaches, options such as distant port placement or floating docking are frequently employed. Assistant roles remain limited in these procedures as well, necessitating greater reliance on intraoperative adjuncts like flexible irrigation systems and operator-managed suction devices.

The learning curve for SP robotic surgery has been systematically evaluated by Pellegrino et al., who analyzed 387 SP procedures performed by a single experienced surgeon, including SP RARP, SP RARN, and SP RAPN [50]. Their findings revealed that significant improvements in outcomes—such as reduced complication rates, operative time, and PSM—occurred only after reaching defined procedural volumes. Specifically, approximately 70 SP nephrectomies and 150 SP prostatectomies were required to meaningfully reduce complications and increase consistency [50]. Minor events such as hematomas and urinary tract infections declined earlier, but major complications, readmissions, and prolonged operative times required more extensive experience to improve. These findings suggest that even seasoned robotic surgeons face a steep learning curve when transitioning to SP systems and underscore the importance of procedural repetition and adaptation. The authors emphasized the importance of a stepwise implementation strategy, including simulation-based learning, dry-lab exercises, and structured mentorship. Institutions seeking to adopt the SP platform should establish clear educational pathways to ensure patient safety and optimize outcomes during the initial implementation period.

Pellegrino’s data—indicating that approximately 70 SP nephrectomies and 150 SP prostatectomies are required to reduce perioperative complications—offer a quantitative anchor for defining milestones in training programs. Translating these findings into practice suggests the need for structured, modular training frameworks, where surgeons progress through phases such as docking proficiency, dissection of key anatomical structures, and reconstruction. Simulation-based training plays a central role in this process. For example, intensive hands-on VR training using platforms like the Da Vinci Skills Simulator has demonstrated significant improvements in robotic skills with statistically significant gains in performance scores (e.g., 69 to 87 for ring walk; *p* < 0.0001) [51]. Beyond conventional simulators, AI-powered educational tools are rapidly emerging. AI-integrated platforms offer personalized, real-time feedback and skill assessment, enabling tailored practice and deliberate improvement [52,53]. These systems have shown promise in video labeling, real-time performance analytics, and adaptive learning environments in robotic surgical training. Together, these strategies—structured training, simulation-based learning, and AI-augmented feedback—can help shorten the SP learning curve while maintaining patient safety and procedural integrity.

## 4. Innovations and Future Perspectives

The field of robotic surgery is undergoing a dynamic transformation, with single-port (SP) systems emerging as a pivotal advancement in minimally invasive urologic oncology. While SP technology is still in its early stages of widespread adoption, ongoing innovations in robotic engineering, data integration, and surgical technique continue to redefine its potential. Technological refinement remains central to future progress. Current limitations in triangulation, retraction, and haptic feedback are expected to be addressed by the next generation of SP robotic platforms. Anticipated improvements include enhanced wristed instruments, better ergonomics, and potentially the reintroduction of missing functionalities such as vessel sealing and fluorescence imaging (e.g., Firefly^®^). These upgrades would allow for greater precision, especially in complex dissections or reconstructive steps such as renorrhaphy and lymphadenectomy. Several institutions have already pioneered novel techniques made possible by the SP platform. For example, the transvesical radical prostatectomy and the Supine Anterior Retroperitoneal Access (SARA) for nephrectomy illustrate how creative anatomic access routes can be facilitated by the platform’s compact design [11,44]. As surgeon familiarity grows, these applications may become more widespread and standardized.

Another transformative innovation is the integration of augmented reality (AR) and preoperative imaging fusion into SP systems. Real-time overlay of multiparametric MRI or CT angiography could enhance anatomical guidance, particularly during nerve-sparing, tumor enucleation, or vascular control [54]. Likewise, robotic vision systems enhanced with AI-based recognition tools may assist in tissue identification and improve surgical safety margins. Artificial intelligence (AI) and machine learning are poised to become core elements of SP robotic surgery. In the operating room, AI could provide real-time feedback, predict complications, or guide the surgical sequence through motion analysis and historical data comparison. Outside the OR, AI-driven virtual simulators are expected to personalize training pathways, monitor technical competency, and help overcome the SP learning curve through adaptive feedback [54].

Telesurgery—once a futuristic concept—has re-emerged as a credible extension of robotic innovation, thanks to the advent of ultra-low-latency networks such as 5G. Recent clinical studies, particularly in China, have demonstrated the feasibility of SP and MP telesurgeries across distances exceeding 6000 km, including complex urologic procedures like radical prostatectomy and cystectomy, with no intraoperative complications and latency consistently under 200 milliseconds [55,56,57]. Robotic platforms such as MicroHand (Shandong WEGO Surgery Robot Co., Ltd., Weihai, China) Edge MP1000 (Shenzhen Edge Medical Technology Co., Ltd., Shenzhen, China), Toumai^®^ (Shanghai MicroPort MedBot (Group) Co., Ltd., Shanghai, China), and KangDuo (Suzhou KangDuo Robot Co., Ltd., Suzhou, China) have been central to these advances, offering dual or triple-console setups, real-time collaboration, and latency-resistant interfaces [58,59]. These systems not only highlight technical feasibility but also hint at the potential of telesurgery to bridge care disparities between high-volume centers and remote or underserved regions. Nevertheless, issues related to cybersecurity, credentialing, cost-effectiveness, and tactile feedback must be resolved before routine adoption.

Beyond innovation, the widespread adoption of SP robotic surgery will ultimately depend on issues of cost-effectiveness and accessibility. SP systems currently represent a substantial financial investment, with acquisition and maintenance costs exceeding those of established MP platforms. These economic considerations are particularly critical in low- and middle-income countries, where limited healthcare budgets and competing priorities may restrict access to robotic surgery. Furthermore, the concentration of SP technology in high-volume centers raises questions of global equity in cancer care. Potential strategies to mitigate these challenges include cost-reduction through competition and market maturation, development of modular or hybrid systems, and the creation of regional centers of excellence that can serve as referral hubs. Future research should not only explore clinical outcomes but also address cost-utility and health-economic analyses to ensure that the benefits of SP technology can be extended across diverse healthcare settings.

On a broader scale, future developments must prioritize global accessibility and health equity. Modular, cost-effective, and potentially portable robotic systems could help democratize access to SP technology, especially in resource-limited settings. The experience from mobile or hybrid robotic systems in low- and middle-income countries could inform design criteria and implementation models that balance innovation with feasibility. Crucially, long-term oncologic and functional outcome data are needed to validate the clinical benefits of SP robotic surgery. Real-world evidence from prospective registries and multicenter collaborations will help define optimal indications, patient selection criteria, and procedural standards. Comparative studies between SP and MP approaches across various urologic malignancies will further inform the integration of SP systems into mainstream practice.

In summary, the future of SP robotic surgery in urologic oncology is poised at a pivotal juncture. As hardware evolves, AI and AR tools mature, and procedural data accumulate, SP systems may redefine what is possible in minimally invasive cancer surgery. The challenge ahead lies not only in technical refinement but also in expanding training, access, and evidence to support its safe and effective global implementation.

## 5. Conclusions

Single-port robotic systems represent a significant advancement in minimally invasive urologic oncology, offering improved cosmesis, reduced invasiveness, and novel access routes. Comparative data suggest that single-port surgery is a feasible and safe alternative to multi-port platforms, with similar perioperative and oncologic outcomes in carefully selected patients (low-complexity tumors, no prior abdominal surgery). However, the single-port approach introduces unique technical challenges and a steep learning curve, requiring tailored training and institutional commitment. Future progress will depend on ongoing technological refinements, the integration of AI, augmented reality and telesurgery, and the accumulation of long-term evidence. As experience grows and systems evolve, single-port platforms are poised to play an increasingly central role in the future of robotic urologic surgery.

## Figures and Tables

**Table 3:** Key studies on single-port robotic radical cystectomy.

Author	Year	Journal	Description	Study Design	N	Key Findings
Kaouk et al. [45]	2019	BJU International	SP-RARC with intracorporeal diversion	Prospective case series	4	SP-RARC with PLND and ileal conduit was feasible and safe in 4 patients, with no conversions, negative margins, acceptable blood loss, and discharge by day 5.
Zhang et al. [46]	2020	Translational Andrology and Urology	SP-RARC with intracorporeal diversion	Prospective case series	4	SP-RARC with intracorporeal ileal conduit was feasible and safe in 4 patients, with mean operative time 270 min, EBL 250 mL, mean nodal yield 12.5, LOS ~5.5 days, and only one Clavien II complication.
Gross et al. [47]	2021	Journal of Endourology	SP vs. MP-RARC comparison	Retrospective cohort	96	In a matched analysis (12 SP vs. 24 MP RARC), SP was feasible with comparable operative time, EBL, complications, readmissions, and PSM rates, though lymph node yield was lower (11.9 vs. 17.1).
Ali et al. [48]	2022	Journal of Endourology	SP vs. MP-RARC with diversion	Prospective cohort	34	In 34 patients (20 MP vs. 14 SP RARC with ICUD), outcomes were broadly comparable; SP was associated with less narcotic use and faster bowel recovery, while operative times and complications were similar.
Fang et al. [49]	2024	Journal of Endourology	SP vs. MP-RARC with diversion	Retrospective cohort	36	In 96 patients (47 SP vs. 49 MP RARC), SP was associated with shorter operative time and faster bowel recovery, while LOS, narcotic use, complications, readmissions, and oncologic outcomes were similar.

**Table 4:** Comparative outcomes between single-port (SP) and multi-port (MP) robotic surgery in urologic oncology.

Domain	Findings (SP vs. MP)	Notes/Explanations
Perioperative outcomes	Similar operative times after learning curve; some reports of modestly reduced blood loss with SP	Early SP series often showed longer operative times due to learning curve; differences narrow with experience
Oncologic efficacy	Comparable oncologic control (positive surgical margins, recurrence, survival); slightly lower lymph node yields in SP	Lower nodal yields reflect surgeon planning, learning curve, risk stratification, and approach (extraperitoneal vs. transperitoneal), not platform limitation
Postoperative recovery	Shorter hospital stay, earlier catheter removal, and reduced opioid use frequently reported with SP	Variability depends on institutional discharge criteria and ERAS protocols; earlier recovery more pronounced in extraperitoneal/transvesical SP approaches
Complication rates	Overall complication rates comparable between SP and MP platforms	Most differences minor and concentrated in early learning curve; major complications similar across platforms

## Data Availability

Data sharing is not applicable.
